# 5G-Enabled Distributed Intelligence Based on O-RAN for Distributed IoT Systems

**DOI:** 10.3390/s23010133

**Published:** 2022-12-23

**Authors:** Ramin Firouzi, Rahim Rahmani

**Affiliations:** Department of Computer and Systems Sciences, Stockholm University, 16407 Stockholm, Sweden

**Keywords:** IoT, distributed intelligence, federated learning, reinforcement learning, fifth-generation mobile network (5G), O-RAN

## Abstract

Edge-based distributed intelligence techniques, such as federated learning (FL), have recently been used in many research fields thanks, in part, to their decentralized model training process and privacy-preserving features. However, because of the absence of effective deployment models for the radio access network (RAN), only a tiny number of FL apps have been created for the latest generation of public mobile networks (e.g., 5G and 6G). There is an attempt, in new RAN paradigms, to move toward disaggregation, hierarchical, and distributed network function processing designs. Open RAN (O-RAN), as a cutting-edge RAN technology, claims to meet 5G services with high quality. It includes integrated, intelligent controllers to provide RAN with the power to make smart decisions. This paper proposes a methodology for deploying and optimizing FL tasks in O-RAN to deliver distributed intelligence for 5G applications. To accomplish model training in each round, we first present reinforcement learning (RL) for client selection for each FL task and resource allocation using RAN intelligence controllers (RIC). Then, a slice is allotted for training depending on the clients chosen for the task. Our simulation results show that the proposed method outperforms state-of-art FL methods, such as the federated averaging algorithm (FedAvg), in terms of convergence and number of communication rounds.

## 1. Introduction

Industrial automation is changing dramatically due to the advent of the Internet of Things (IoT) in industrial applications. This change is occurring due, in part, to the recent technological advancements which allow resource-constrained devices (e.g., smart sensors, activity trackers, etc.) to collect data from the environment without human invention. According to Statista, the number of IoT-connected devices will reach 30 billion by 2030 [1]. Despite its rapid expansion, the IoT still faces major computational, storage, and networking challenges [2]. Most of these are mainly due to the centralized structure of the current IoT architecture.

Initially, due to the limited computing power of resource-constrained devices, data had to be sent to a server with enough computing power to process these vast amounts of data. However, it became clear that sending large amounts of data over the network to a server is not practical due to bandwidth and privacy constraints. Thus, distributed IoT architecture has aroused researcher interest as an alternative to the outmoded centralized paradigm and which may be able to revolutionize the IoT infrastructure [2]. 

Edge computing has recently acquired appeal as the underpinning technology for distributed intelligence [3]. Edge computing is a more natural way to process data at the edge of the network rather than sending it to a centralized cloud. The aim with edge computing is to bring computing power closer to where devices consume or generate data, providing ultra-low latency and real-time access to network data that can be used by various applications. Application and decision-making latency are lowered by putting computation close to the edge. Faster answers and actions result from less back-and-forth movement from the edge to the core.

Implementing edge computing is not straightforward. Network bandwidth shifts as enterprises transfer computation and data storage to the edge. Enterprises have traditionally allocated more bandwidth to data centers and less to endpoints. The demand for greater bandwidth throughout the network is being driven by edge computing. Therefore, edge computing is frequently mentioned together with the fifth generation of public mobile networks (so-called 5G). Although these two technologies are not interdependent, combining them can efficiently address many of the challenges of both. 

In 5G, delay and network bandwidth limitations are overcome. As a result, work may be concentrated on tackling the performance and efficiency concerns associated with edge computing. At present, industries such as manufacturing, ports, mining, airports, energy, and healthcare can have their own private 5G networks.

A private 5G network [4] is a local area network that provides dedicated wireless connectivity inside a defined area. Most notably, it may be administered independently by its owner, who controls the network in every aspect, including resource allocation, priority scheduling, and security [4]. Industry users can define and specify their own security strategies and rules (e.g., keeping sensitive data locally). The major drawback of Ethernet compared to 5G is its use of expensive and bulky wired equipment. 5G, by getting rid of these limitations, can connect many constantly moving devices. Private 5G networks provide ultra-low latency, massive connectivity, spectrum flexibility, wider bandwidth, and ultra-high reliability.

Therefore, 5G is moving away from the limitations of current cellular deployment approaches, in which hardware and software are coupled, toward programmability, openness, resource sharing, and edgefication to implement connectivity-as-a-service (CaaS) technologies, such as private cellular networking.

The state-of-art networking principles (e.g., virtualization, multi-access edge computing, SDN) used by 5G make network management dynamic and agile, both at the core and the edge of network [5]. This advantage gives 5G the power to effectively handle a large number of IoT devices and their communications and to also be able to deliver the required bandwidth, which leads to the achievement of ultra-low latency. In parallel, recent advances in radio access network (RAN) technologies, such as the software-defined paradigm, have made it possible to decouple software from hardware, ending the monopoly on radio access networks and allowing researchers in academia and industry to have access to real cellular networks. Thus, the way researchers and the telecom industry develop, install, and interact with cellular networks has been fundamentally altered due to these new software and hardware components.

Today, software packages such as OpenAirInterface (OAI) [6] and srsLTE [7] have simply embraced the software-defined paradigm popularized by the GNU radio libraries [8], allowing rapid instantiation of fully functional cellular networks on commercial software-defined radio (SDR) devices. 

On the way to achieving the openness of mobile networks, O-RAN was created in 2018. O-RAN [9] is a software architecture that runs on disaggregated software-based virtualized components that communicate with each other through a set of standardized interfaces. Using open, standardized interfaces allows operators to integrate equipment from a variety of manufacturers as well as lets smaller companies participate in the RAN ecosystem. To put it another way, infrastructure sharing not only increases resource usage but also creates new market opportunities (e.g., differentiated services, infrastructure leasing, and CaaS), making it an appealing option for both network operators and infrastructure providers [5].

According to a preliminary investigation, O-RAN, mobile edge computing (MEC), and network slicing (NS) are substantially connected, partially complimentary, and partly overlapping. Furthermore, some of the techniques invented by one can be effectively reused by the other [10]. On the other hand, their direct integration may result in high complexity, compromising overall system performance. It would therefore be beneficial if they could be appropriately integrated with a new decomposition and redundant functional blocks removed to offer overall synergy.

In this paper, we propose an optimization method for federated learning (as distributed intelligence) based on edge computing in 5G to implement the three-layer architecture of “client-edge–cloud”, where local model parameter updates are performed on the “client-edge”, and global aggregation is performed between the “edge–cloud”. The following are the key advantages of our framework:

- First, by formulating an optimization problem to balance the performance and cost of federated learning, we provide a framework for deploying federated learning in 5G systems through O-RAN. In particular, we focus on leveraging O-RAN intelligent controllers to optimize and improve the performance of FL;
- Second, a reinforcement learning model is proposed for client selection in each FL task and resource allocation to perform model training in every iteration;
- Finally, the numerical simulation and experiment results are provided to evaluate the performance gains. Compared with existing, optimized federated learning schemes, the results show that our algorithm can effectively balance accuracy and cost.

The rest of the paper is organized as follows. Section 2 presents the background and gives an overview of related works. Section 3 describes the proposed model’s main characteristics and the system’s goals in detail. In Section 4, we perform a performance evaluation and highlight the results obtained from the experiments. Finally, Section 5 presents our conclusions.

## 2. Background and Related Work

### 2.1. Federated Learning 

Over the past decade, the use of distributed machine learning to enable intelligent IoT services at the network edge has been inevitable. As a promising paradigm for distributed learning, federated learning has become a popular research area. Federated learning was first introduced by McMahan et al. [11]. Federated stochastic gradient descent (FedSGD) is its vanilla algorithm, where clients locally train a model with SGD in each round and then upload it to the server for aggregation. Use of FedSGD can dramatically mitigate privacy concerns while achieving the same accuracy as centralized model training for IID data.

Meanwhile, due to the frequent uploading of models in federated learning, there may be a communication overhead that leads to a decrease in efficiency. Therefore, McMahan et al. [12] proposed an improved algorithm version called FedAvg, where clients can synchronously execute multiple rounds of SGD in each training round (before uploading the model to the server). This leads to fewer rounds of communication and higher effectiveness of federated learning.

In [13], the authors focused on the problem of selecting clients with resource constraints to reduce communication time. They proposed a protocol that assigns clients a deadline for downloading, updating, and uploading ML models. Then, the MEC operator chooses clients such that the server may combine as many client updates as it can in a finite amount of time, improving the efficiency of the entire training process and cutting down on the amount of time needed to train ML models.

Zhao et al. [14] discovered that the performance and efficiency of federated learning, especially FedAvg, drop sharply when the data are non-IID. The presence of non-IID data in federated learning is due to the random selection of clients. The data may differ significantly between various clients. Therefore, by selecting a random subset of clients, the true data distributed for a global model may not be accurately represented.

Wang et al. [15] studied the federated learning convergence bound from a theoretical perspective. To minimize the loss function within a given resource budget, they proposed a control algorithm that identifies the appropriate tradeoff between local updating and global parameter aggregation.

Sattler et al. [16] proposed an adapted sparse ternary compression (STC) framework to reduce the communication cost for federated learning in the presence of non-IID data. Compared with other compression methods, their proposed STC protocol is superior since fewer gradient evaluations and transmitted bits are required to converge to a given goal accuracy.

In another study, Wang et al. [17] analyzed the connection between the distribution of training data on a device and the trained model weights based on that data. They proposed a reinforcement learning (RL) system that uses deep Q-learning and learns the correlation between the distribution of training data and model weights to select a subset of devices in each communication round to maximize the reward, thereby promoting increased validation accuracy and penalizing the use of more communication rounds. In the designed system, each state of the RL agent corresponds to a weight of the DNN model, and, as the authors mentioned, the state space of the RL agent can be huge, since a deep learning model can have millions of weights. They applied a lightweight dimension reduction method on the state space.

Zhang et al. [18] addressed the aforementioned challenge by developing an efficient federated learning framework leveraging multi-agent reinforcement learning (FedMARL), concurrently improving model accuracy, processing latency, and communication efficiency. In their research, the agent reward was defined by a linear combination of accuracy, latency, and communication efficiency; therefore, the agent state space was noticeably smaller. However, FedMARL is not preferred for circumstances where computing is limited, since it may raise the computational load of nodes. 

Although research works have studied the enhancement of federated learning performance in statistical heterogeneity settings, they have encountered issues with increased processing and communication loads as well as challenges relating to practical application.

Therefore, there is a need to explore a superior strategy that may mitigate or resolve the problems mentioned earlier while maintaining or enhancing performance.

### 2.2. O-RAN 

Like other computer science and communication areas, RAN technology is moving from monolithic to microservice architecture. In this context, the O-RAN consortium was established in 2018. The two main goals of this consortium are to separate RAN functions and provide standard interfaces for functions to communicate with each other [19]. This helps telecos to avoid being locked into a specific vendor. It also allows an operator or system vendor to assemble “best-of-breed” network components from an ecosystem of multiple vendors.

This technology also enables artificial intelligence to be embedded in all RAN management layers through controllers embedded at the edge of the network [20]. Since the architecture of O-RAN follows the microservice architecture, and its interfaces are carefully designed and standardized, it is easy to replace the implementation of the components with more efficient implementation. In such a design, this possibility is quickly provided by using off-the-shelf services. 

**Figure 1:**
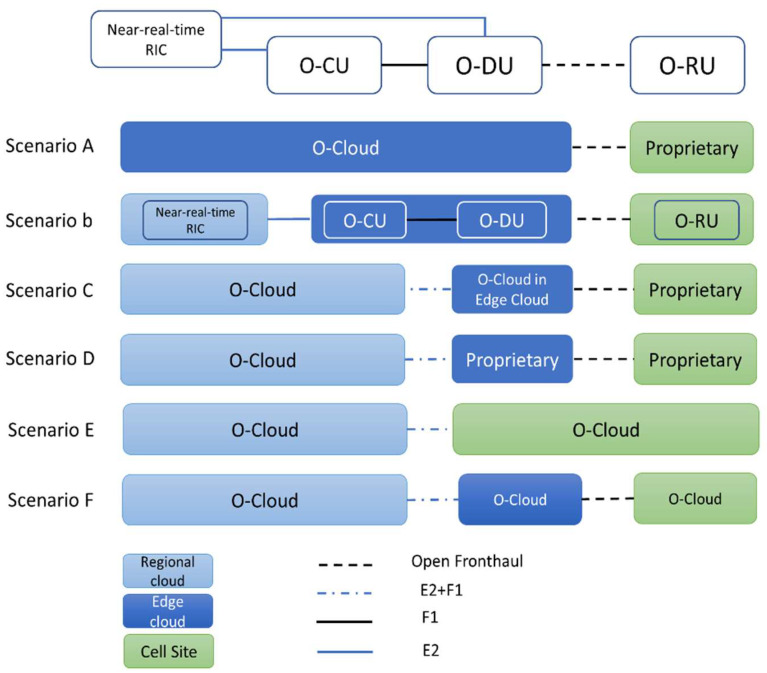
O-RAN deployment scenarios.

Figure 1 illustrates six envisioned O-RAN deployment scenarios. The difference between these scenarios is where the virtual network functions (VFNS) are deployed. Two main places to implement these functions are O-Cloud and proprietary cell sites. The O-Cloud can be any hardware capable of independently running the O-RAN software, for example, edge cloud and regional cloud. Thanks to O-RAN’s standard interfaces, O-RAN’s software can be run anywhere, including outside the proprietary cell site, regardless of the hardware.

As shown in Figure 1, one could argue that Scenario A is the simplest scenario in terms of architecture, wherein all components except for the RUs are deployed in the O-Cloud. However, in Scenario B, where the RIC is deployed in the regional cloud, the CUs and DUs are at the edge, and the RUs are at the cell sites as the predominant deployment option [21]. In this paper, we implement this scenario in our test bed.

## 3. Proposed Approach

To develop a distributed intelligence system for the IoT in the 5G environment, in this paper, we propose an architecture consisting of two parts: a high-level and a low-level part. The low-level part of the architecture focuses more on the network overlay. It is proposed to implement a distributed intelligence network overlay using the 5G and O-RAN components mentioned in the previous sections. The high-level part of the architecture focuses more on the software components needed to create the distributed intelligence system scattered across the network overlay.

### 3.1. Low-Level Architecture 

In this study, we employ O-RAN as the underlying architecture that includes multiple network functions and multiple slices for downlink transmission. Moreover, we take advantage of the two radio intelligent controllers (RICs): a near-real-time RIC and non-real-time RIC. The near-RT RIC is suitable for operations that can tolerate less than one second of latency, such as hosting third-party applications (xApps) which communicate with the centralized unit (CU) via standard open interfaces and implement the intelligence in the RAN through data-driven control loops. The non-RT RIC is suitable for operations that can tolerate a latency of more than one second, such as training artificial intelligence (AI) and machine learning (ML) models. The O-RAN system includes components other than the RICs: the radio unit (RU), the distributed unit (DU), and the centralized unit (CU). The CU itself consists of a user plan (UP) and a control plan (CP).

**Figure 2:**
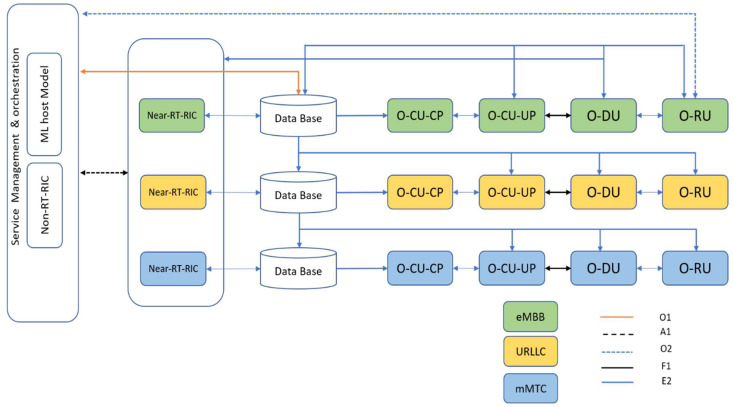
O-RAN setup for underlying architecture.

In 5G, three primary use case classes are introduced in terms of different levels of quality of service (QoS), namely, ultra-reliable low-latency communication (uRLLC), extreme mobile broadband (eMBB), and massive machine-based communication (mMTC) [22]. As shown in Figure 2, there are three dedicated slices for each of the above classes, and each slice may contain several UEs with similar QoS requirements. Through the E2 interface, the data relating to the slice operation are gathered and stored in distributed databases. The O1 interface is used to transfer the data to near-RT RICs, and the A1 interface can be used to transfer the data to the non-RT RICs. 

In this paper we train our reinforcement learning agent in the non-RT RIC controller using the data from the databases and clients. The trained model is deployed in the near-RT RIC to select more appropriate clients for the federated learning training process in a real-time situation. To this end, we use Acumos [23] and ONAP [24], as in [25]. Acumos is an open-source framework for the building and deployment of AI applications. Acumos can facilitate both the training of ML models and the publishing of trained ML models to RIC. ONAP is another open-source platform used to manage, orchestrate, and automate both physical and virtual network functions. It offers the automation and lifecycle management of the services for next-generation networks [25].

The FL learning process needs to be improved, but iteratively training the model brings about the challenge of learning time and latency. Thus, as we discuss in Section 3.2.1, the objective function of our proposed reinforcement learning is to help reduce the learning time while maintaining a high level of accuracy for the global model.

### 3.2. High-Level Architecture 

IoT applications have heterogeneous device, statistic, and model properties that bring about major challenges for vanilla federated learning. Clustered FL (also known as federated multitask learning [26]) can be a realistic approach for addressing such heterogeneity issues. Multitasking allows devices with the same properties to jointly train their customized model.

Therefore, we seek to achieve great flexibility through the development and deployment of reinforcement learning, which allows devices of the same type (e.g., parking lot cameras) to train their models considering their resources and application requirements while taking advantage of federated learning for knowledge sharing.

**Figure 3:**
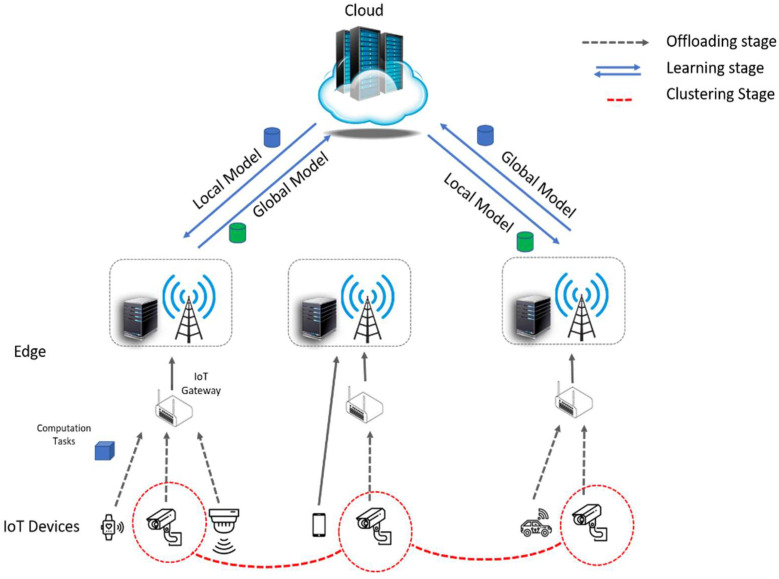
The multitask federated learning framework for intelligent IoT applications.

To this end, we propose a multitask federated learning architecture for intelligent IoT applications. Our proposed system leverages a cloud–edge design, as shown in Figure 3, which places essential, on-demand edge processing resources near IoT devices. To support highly efficient federated learning for intelligent IoT applications, we use reinforcement learning to group IoT devices based on the type and volume of their data and latency. This method allows devices with the same characteristics to jointly train a shared global model by aggregating locally computed models at the edge while keeping all the sensitive data local. Thus, the requirements of IoT applications for high processing efficiency and low latency can be met.

As shown in Figure 3, the collaborative learning process in our proposed approach mainly includes the following three phases:

- Offloading phase: The IoT devices can offload their entire learning model and data samples to the edge (e.g., edge gateway) for fast computation. The devices can also split the model into two parts and keep the first part and train it locally with their data samples and offload the other part of the model to the edge for collaborative data processing [27]. In this work, we assume that IoT devices either perform the whole training model or offload it to the edge (IoT gateway);
- Clustering phase: To capture the specific characteristics and requirements of a group of devices, we propose a reinforcement learning method to cluster devices based on the distribution of their data, size of data, and communication latency. The mechanism of reinforcement learning is elaborated on in the next section;
- Learning phase: After uploading data to the IoT gateway, the local model is trained and then transmitted to the master node. The master node averages the parameters of received local models into a global model and sends it back to the edges. This training procedure is repeated until the expected accuracy is achieved. As a result, a high-quality global model is obtained.

#### 3.2.1. Reinforcement Learning (RL) System Design

The problem of FL client selection is challenging to address without a sample of client data. We, instead, model the situation as an RL problem. In the first round of FL model training, we choose the client randomly afterward; in the second round, the RL agent decides which client is selected for the training by analyzing their training loss and communication status. In this section, we present our problem formulation and RL system design. We define our optimization target to reduce the latency and learning time while maintaining a high level of global model accuracy as follows:

(1) $$\underset{A}{\max}\left[Acc\left(T\right)-H_{T}-L_{T}\right]$$

where *Acc*(*T*) is the accuracy of the global model after the training process on the test dataset, $H_{T}$ is the sum of the training time of all training rounds, $L_{T}$ is the sum of the latency of all training rounds, and *A* is the set of actions which form the matrix for client selection.

##### Agent Training Process

Each global model in our proposed system is assigned to an RL agent at the central server. The RL agent makes a decision about clients that can participate in the process of model training. The agent takes action $\alpha_{n}^{t}$ based on its current state. The agent’s reward is computed based on the test accuracy improvement on the global model, processing time, and communication latency.

##### Design of RL Agent States

Accuracy, training time, communication latency, baseline training time for each device, and the current training round index are the four parameters that form the RL agent’s input state. At each round *t*, the agent selects 10 out of 15 clients to participate in the training process based on the input state, and the reward is then computed based on the decision. 

Equation (1) shows the input state and output action at round *t*.

(2) $$\begin{array}{c} \;S^{t}=\left\{L_{t}^{n},H_{t}^{n},\;Acc_{t}^{n},\;B^{n},t\right\}\\ a^{t}\in \;\left[0,1\right] \end{array}$$

where *S^t^* is the input state for the agent at round *t*, $L_{t}^{n}$ is the latency of training round *t* for device *n*,$\;H_{t}^{n}$ is the training time of training round *t* for device *n*, $Acc_{t}^{n}$ is the accuracy of the global model after the training process of round *t* for device *n*, *t* is the current round of training, $B_{t}^{n}$ is baseline training time for device *n*, and $a^{t}$ is the output action that indicates whether a client is terminated after the training process of the current round.

##### Design of the Reward Function 

The reward function should account for changes in test accuracy, processing time, and communication latency in order to maximize FL performance. The client with the longest training period decreases the reward and may not participate in the training process. Therefore, to calculate a fair reward, a normalizing function (*f_norm_*) is employed. The training time for each device when the data size is relatively sufficient to reach the target accuracy is denoted as *B^k^* (a baseline). The training time of device *k* ($H_{t}^{k}$) is normalized according to *B^k^* using Equation (3).

(3) $$f_{norm}=\{\begin{array}{c} 1-\frac{H_{t}^{k}}{B^{k}}\;\;\;\;H_{t}^{k}\leq \;B^{k}\;\\ \frac{B^{k}}{H_{t}^{k}}-1\;\;\;\;H_{t}^{k}>B^{k}\; \end{array}$$

The reward *r_t_* at training round *t* is defined as:

(4) $$r_{t}=\left[U\left(Acc\left(t\right)\right)-U\left(Acc\left(t-1\right)\right)\right]-f_{norm}(H_{t})-L_{t}$$

where Acc(t) represents the accuracy of the model in round *t*, $H_{t}$ denotes the total training time in round *t*, and $L_{t}$ is the total communication latency for round *t*. During the final rounds of the FL process, the utility function *U*(.) ensures that *U*(*Acc*(*t*)) can still change somewhat even if the improvement in *Acc*(*t*) is small.

##### Choice of Algorithm

The RL agent can be trained to achieve the goals outlined in Section 3.2.1 using several algorithms; examples are REINFORCE [28] and DQN [29]. Proximal policy optimization (PPO) [30] is utilized in this study. It is a cutting-edge technique that is comparatively simple to apply and performs well on common RL benchmarks [30]. Additionally, PPO generates the output as an explicit action by utilizing a policy network [30] in contrast to DQN, which calculates the best action by analyzing all potential actions using the Q-network [30]. PPO can be used for both environments with discrete and continuous actions. PPO belongs to the category of off-policy RL algorithms, which repeatedly use data from interactions between the agent and the environment (explorations) [27]. Although exploration is time consuming, this improves training efficiency. Thus, we select PPO as the RL algorithm.

##### RL Training Methodology

**Figure 4:**
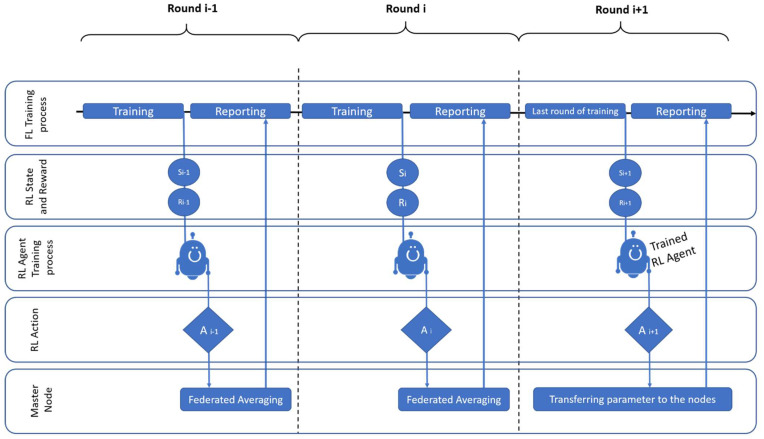
Training of the RL agent.

The RL agent contains two fully connected networks, critic and actor, each of which has three layers. The actor picks an action to observe, and the critic network then predicts the agent reward for the specific action. In the first step, it samples from the most recent iteration of the stochastic policy to compile a collection of trajectories for each epoch. The next step is to update the policy and fit the value function by computing the rewards-to-go and the advantage estimations. A stochastic gradient ascent optimizer is used to update the policy, and a gradient descent technique is used to fit the value function. Until the environment is solved, this process is repeated for several epochs. If we want to train the agent online, then the agent must wait until the end of each training round to obtain the training time to calculate the reward, so the best option is to train the agent offline. The learning process of the agent is shown in Figure 4.

## 4. Results

In this section, we first describe the experimental setup and evaluate the performance of our proposed approach.

### 4.1. Experimental Setup

As a proof-of-concept scenario to demonstrate that the proposed approach performs effectively, a private 5G network was implemented. The core network was implemented using Magma [31] and RAN using O-RAN software version E, which was developed by the O-RAN software community (OSC). Three types of IoT devices were used:

- Raspberry Pi 4 Model B with 1.5 GHz quad-core ARM Cortex-A72 CPU 4 GB RAM;
- Raspberry Pi 2 Model B with 900 MHz quad-core ARM Cortex-A7 CPU, 1 GB RAM;
- Jetson Nano Developer Kit with 1.43 GHz quad-core ARM A57, 128-core Maxwell GPU and 4 GB RAM.

We evaluated our proposed approach by training visual geometry group (VGG) networks [32]. A VGG is a standard convolutional neural network (CNN) architecture and has several layers. In this paper, we used two version of it, namely, VGG-5 (five layers) and VGG-8 (eight layers), each of which has three fully connected layers, and the rest are convolutional layers. The CIFAR-10 dataset was used, and a batch size of 100 was used for all experiments. There were 10 K testing samples and 50 K training samples in the dataset. On the server, the FedAvg aggregation method was applied.

To verify the effectiveness of our proposed approach, we examined several non-IID data levels. We compared our proposed approach with FedAvg performance as benchmarks. As in [12], the number of devices that were chosen in each round, K, was fixed to 10.

In order to simulate non-IID data, we defined four different classes, as in [17]:

- Class-1: All the data on each device have one label;
- Class-2: Indicates that data on each device evenly belong to two labels;
- Class-5: 50% of the data are from one label, and the remaining 50% of data are from other labels;
- Class-8: 80% of the data are from one label, and 20% of data are from other labels;

### 4.2. Performance Metrics

The performance metrics for the evaluation of our proposed system are as follows.

#### 4.2.1. Number of Communication Rounds

In federated learning, due to the limited computation capacity and network bandwidth of IoT devices, reducing the number of communication rounds is crucially important. Thus, we used the number of communications rounds as the performance metric of our proposed approach.

#### 4.2.2. Accuracy

Accuracy is defined as the number of correct predictions divided by the total number of predictions. This metric measures how accurate a new aggregated model is against the test data in each FL round. Although the data are highly unbalanced in the two classes, we used accuracy instead of well-known metrics for binary datasets such as precision, recall, and F1 score, since, in this study, we focused on the selection of more appropriate clients for better FL accuracy under the non-IID settings. In [12,14], where researchers carried out similar work, it was shown that accuracy is the primary FL performance metric in the non-IID settings.

### 4.3. Experimental Results

**Figure 5:**
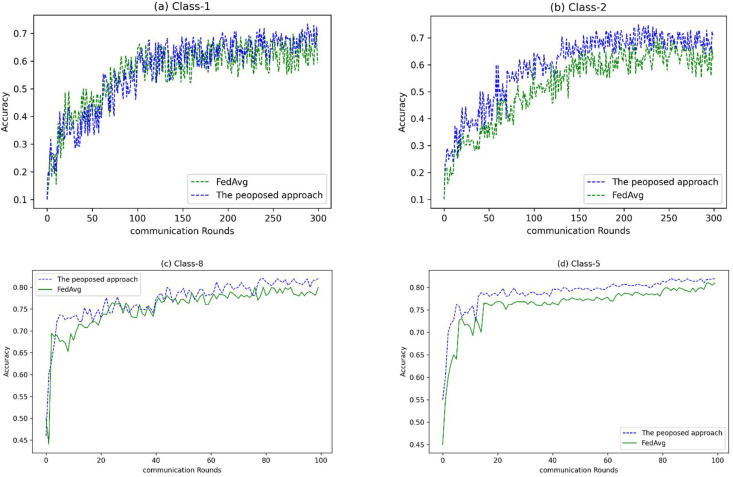
Accuracy for different number of communication rounds on various CIFAR-10 non-IID levels (VGG-5 DNN).

To evaluate the proposed system, we compared it with the well-known federated learning algorithm FedAvg. As mentioned in the previous section, this comparison was performed for different levels of non-IID data with two deep convolutional networks. Figure 5 shows the accuracy of VGG-5 on different levels of non-IID data. For the training process, 10 out of 15 clients were used in each round. There were 20 epochs of training in each round.

As can be seen in Figure 5a, at this level of non-IID data, where all the data belong to one label, although the proposed method was less accurate in the initial rounds of training, it reached higher accuracy than FedAvg in the last rounds of the learning process. What is clear at this level of non-IID data is that the learning curve for both algorithms has erratic fluctuations, which is normal.

For Class-2 non-IID data, where the data belong evenly to two labels, the proposed algorithm was more accurate from the beginning of the training process to the end, as shown in Figure 5b.

For Class-8 and Class-5, Figure 5c,d, the proposed algorithm achieved higher accuracy than the FedAvg algorithm from the beginning to the end of the process. It is worth mentioning that FedAvg was able to achieve the same accuracy as the proposed algorithm but with more rounds of training.

What can be seen in Figure 5 is that our proposed method was more accurate than the FedAvg method for all levels in the initial training rounds, except for class one, where this superiority occurred at the end of the training period. It is worth mentioning that, for this level of non-IIDness, where the type has the highest degree of non-IIDness, it was not expected that a method could be superior with a good margin.

In the second experiment, VGG-8 was trained using trained RL for VGG-5. The results show that the proposed approach again had a better performance than FedAvg. For the two CNN models on CIFAR-10, each entry in Table 1 indicates the number of communication rounds required to obtain the targeted accuracy (indicated in parentheses). 

Comparison of the results presented in Table 1 validates that, in all levels of non-IID data, the proposed method needs fewer training resources to achieve the desired accuracy, which can greatly help IoT applications in the 5G environment in terms of saving resources and also reducing latency.

It may be concluded from the comparison of results presented in Table 1 that, to save resources and time, the proposed RL approach can be trained using shallower networks first (VGG-5) in a non-RT RIC and then deployed in a near-RT RIC to train deeper FL networks (VGG-8).

Although FedAvg is designed explicitly for federated learning environments, performance degrades sharply in the presence of data that are non-IID. This finding is in line with the findings of Zhao et al. [14], who showed that, in contrast to IID learning settings, model accuracy can decrease by up to 55% in non-IID learning contexts. This effect can be worse if the system’s settings are highly distributed, and each client has only a tiny portion of the data. Our experiments have already shown that the degree of IIDness of local client data significantly affects the convergence behavior of federated averaging. In contrast, our proposed method appears to be more reliable. Our proposed method attempts to mitigate the non-IIDness problem by selecting appropriate clients with similar data distribution and volume. In addition, our method considers latency, a significant factor in the latest generation of mobile networks.

As mentioned in Section 2, similar studies have been conducted to optimize the selection of federated learning networks using RL, but what distinguishes this work and its results from others is the system design based on the requirements and characteristics of IoT systems in the 5G/6G environment.

## 5. Conclusions

Due to restricted control over the learning environment, implementing FL in mobile networks and IoT is challenging. This paper uses the O-RAN architecture to deploy and optimize FL as a distributed intelligence method for distributed IoT networks. We proposed a client selection method based on RL to solve the problem of ensuring optimal device selection and resource allocation decisions online. Simulation results based on real-world datasets demonstrated that the proposed method can achieve high FL accuracy while meeting latency requirements. The optimized FL model outperforms state-of-the-art FL methods in terms of the number of communication rounds, latency, and accuracy. Therefore, the client selection method can be used in control loops of O-RAN to ensure the selection of the most appropriate clients for training. In the future, we will investigate the location of the distributed data collection points to improve our method in a highly distributed environment.

## Figures and Tables

**Table 1:** The number of communication rounds required to obtain the targeted accuracy.

Algorithm	Non-IDD	VGG-5	VGG-8
FedAvg	Class-0	51 (80%)	20 (80%)
FedAvg	Class-1	1474 (78%)	1394 (78%)
Proposed		1198 (78%)	1094 (78%)
FedAvg	Class-2E	294 (70%)	253 (70%)
Proposed		196 (70%)	123 (70%)
FedAvg	Class-5	80 (80%)	66 (80%)
proposed		69 (80%)	53 (80%)
FedAvg	Class-8	203 (80%)	185 (80%)
proposed		101 (80%)	94 (80%)
